# Editorial: Effects of combined EMF exposures and co-exposures, volume II

**DOI:** 10.3389/fpubh.2022.1052639

**Published:** 2022-10-14

**Authors:** Mats-Olof Mattsson, Myrtill Simkó, Maria Rosaria Scarfi, Olga Zeni

**Affiliations:** ^1^SciProof International (Sweden), Östersund, Sweden; ^2^National Research Council (CNR), Rome, Italy

**Keywords:** electromagnetic fields, combined exposures, intermediate EMF, radiofrequency EMF, co-exposure

Research devoted to health risk assessment of exposure to electromagnetic fields (EMF) has sparingly focused on exposures to more than one type of exposure (a combined EMF exposure) and on co-exposures of EMF with other environmental agents. This is probably due to several reasons, including technical considerations as well as the reductionist approach to science, which advocates investigating “one factor at a time.” However, the last decade has seen a growing interest in scientific disciplines such as toxicology and environmental sciences toward understanding effects of simultaneous exposures.

The “real” exposure is multifaceted, and often very complex. Therefore, this Research Topic of Frontiers in Public Health—Radiation and Health intended to put focus on this important topic by providing a forum for original research and analytical overviews, and hopefully generating an increased interest in an issue which is highly relevant for scientists as well as other EMF stakeholders.

Two of the papers in the Research Topic come from the same research group and deal with the exposure assessment to intermediate frequency electromagnetic fields (IF-EMF) in libraries in Japan.

In the paper by Ikuyo et al., exposure to magnetic fields from electronic article surveillance (EAS) gates was evaluated. The transient exposure of people due to passing through or beside the gate, and the chronic exposure of workers in the room due to the long hours in the space surrounding the gate, were studied. As a result of a survey of university libraries in Japan, the two most common EAS gate models were selected for further study. To quantitatively estimate the human exposure, detailed measurements were carried out, and the induced electric field in a human body was numerically calculated for exposures to magnetic fields of the two EAS gate models. Magnetic field distribution was measured in a large room of one gate model to assess the chronic exposure of library workers at their desk. The results indicated that the magnetic field was distributed as a function of the horizontal distance to the nearest gatepost. Authors also identified the 45-point average value B_IEC_ defined by IEC and CENELEC standards as a useful quantity to compare exposures from different EAS gates.

The paper by Yamaguchi-Sekino et al. aimed to assess exposure levels due to a combination of IF-EMF due to the EAS systems and pulsed EMF due to the handling of activator/deactivator of anti-theft tags termed as book check units (BCUs). The exposure scenario was defined based on information regarding the usage of EAS gates and BCU derived from data collected by means of a questionnaire distributed to 4,073 libraries in Japan. Based on the 548 completed questionnaires, four exposure patterns were defined according to various exposure scenarios, and both short-term and mid-term exposures were defined. The results of the measurements indicated that the ICNIRP exposure limit was not exceeded in any case.

The results of these two papers, taken together are useful for future epidemiological studies on the possible health effects due to this specific case of combined EMF exposure.

Jeschke et al. provide a review where they employed an occupational health and safety perspective on the most recent ICNIRP guidelines (1) on EMF in the frequency range 100 kHz−300 GHz. The paper has several parts, of which a comparison with the previous guidelines (2) plays a substantial part. Also, other guidelines, including other frequency ranges are mentioned as they are pertinent for occupational safety and health risk assessment. Furthermore, the paper addresses the rationale in ICNIRPs threshold settings, practical aspects of compliance assessment, and open questions in practical applications as well as identified knowledge gaps. The authors also stress the difference in exposure settings between exposure in the occupational situation's vs. exposure of the general public and point to that other environmental factors as well as non-adverse health effects can influence occupational safety and health.

In an animal study from Yan et al., the authors investigated the possible effects of long-term (10 weeks or longer) RF EMF exposure on the male mice fertility. The exposure of free-running male C57BL/6J mice was to a 2 GHz far-field (2.5 W/m^2^ power density, calculated whole-body SAR 0.125–0.5 W/kg), 3 h/day. The animals were exposed for 10 weeks, followed by a 4 week mating period, and for some of the animals for another 4 weeks. F1 offspring were exposed for 3 weeks. Several endpoints related to reproductive outcomes, testis and sperm morphology and function, and offspring growth and glucose metabolism were investigated. No effects of exposure on male mice fertility were observed, although testis germ cells displayed a higher degree of apoptosis than controls. The male, but not female, F1 offspring had a slower growth rate than controls, and did also exhibit decreases in glucose metabolism markers.

The study did not provide evidence for any effects on male mice fertility, but provides several novel aspects compared to previous studies in the field, which have a number of inconsistent findings.

## Conclusions

In summary, the papers presented in this special issue represent a diverse account of the complex exposure situation around specific devices due to combinations of static MF, IF-EMF, and RF-EMF in occupational settings. Since this topic deserves great attention for the purpose of exposure assessment of relevance for compliance with exposure limits and for its central role within the process of health risk assessment, we hope that the readers can find these articles informative and useful to perform ground-breaking research in this area.

The collection is also adding relevant evidence to the question of possible effects on reproduction from exposures to RF EMF at levels below generally recognized exposure limits. Strikingly, such studies are by far lacking combinatorial or co-exposure components.

We would like to thank the researchers who submitted their contributions. We are also grateful to the reviewers who helped in the evaluation of the manuscripts and made very valuable suggestions to improve the quality of the contributions.

## Author contributions

All authors contributed equally to the conception, drafting, and editing of the manuscript.

## Conflict of interest

Authors M-OM, MS, MRS, and OZ were employed by SciProof International (Sweden) and National Research Council (CNR).

## Publisher's note

All claims expressed in this article are solely those of the authors and do not necessarily represent those of their affiliated organizations, or those of the publisher, the editors and the reviewers. Any product that may be evaluated in this article, or claim that may be made by its manufacturer, is not guaranteed or endorsed by the publisher.
